# The overlooked bacterial pandemic

**DOI:** 10.1007/s00281-023-00997-1

**Published:** 2023-12-11

**Authors:** Danilo Gomes Moriel, Diego Piccioli, Maria Michelina Raso, Mariagrazia Pizza

**Affiliations:** 1 GSK Vaccines Institute for Global Health, Via Fiorentina 1, 53100 Siena, Italy; 2 Imperial College London, London, UK

## Abstract

The COVID-19 pandemic had a significant economic and health impact worldwide. It also reinforced the misperception that only viruses can pose a threat to human existence, overlooking that bacteria (e.g., plague and cholera) have severely haunted and shaped the course of human civilization. While the world is preparing for the next viral pandemic, it is again overlooking a silent one: antimicrobial resistance (AMR). This review proposes to show the impact of bacterial infections on civilization to remind the pandemic potential. The work will also discuss a few examples of how bacteria can mutate risking global spread and devastating outcomes, the effect on the global burden, and the prophylactic and therapeutic measures. Indeed, AMR is dramatically increasing and if the trend is not reversed, it has the potential to quickly turn into the most important health problem worldwide.

## Introduction

Vaccines and antibiotics have substantially changed the course of the history of infectious diseases. Many deadly and devastating infections are now preventable by vaccination and can be cured with antibiotics, resulting in an overall reduction of morbidity and mortality. Vaccines and antibiotics have limited the global dissemination and the risk of pandemics caused by bacterial pathogens, leaving the misperception that only viruses are responsible for the pandemics that have haunted human civilization throughout history. This concept has been recently reinforced by the COVID-19 pandemic, which has caused the death of millions of people worldwide, has disrupted the global economy, and has required the most rapid and coordinated global effort to tackle it.

However, after about 100 years from the discovery of the first antibiotic, antibiotic resistance is increasing to dangerous levels in all areas of the world. The emergence of new resistance mechanisms spreading globally represents one of the major concerns for the global health. Bacteria may acquire resistance to antibacterial drugs through a variety of mechanisms. There are bacteria which are innately resistant, but there are also bacteria that become resistant to an antibacterial agent and proliferate and spread under the selective pressure. It is of great concern the rise in healthcare facilities of bacterial pathogens that express multiple resistance mechanisms (superbugs) which renders the treatment very complex and increases both human morbidity and financial costs. Because of the limited efficacy of antibiotics against superbugs, even the treatment of common infections can become very difficult, and this may represent a risk even during surgeries, or for chemotherapy treatments that are known to reduce immune functions [1]. Antibiotics can also alter the gut microbiome, reduce its diversity [2], and even cause the killing of beneficial microbes, with serious consequences for the host [3]. The concept of resistance has been broadened and includes not only antibiotics but all antimicrobials, such as antiviral, antifungal, and antiprotozoal drugs. Because many microbes can infect not only humans but also other animals and plants, the antimicrobial resistance (AMR) is being considered a “One Health” problem [4].

AMR is particularly of concern in the poorest countries, where civil conflicts, poor hygiene, malnutrition, and shortage of water supplies increase the risk of the rapid spread of infectious diseases and consequently the possible emergence of resistant pathogens. The impact of AMR on public health, measured not only as attributable death but also in terms of disability-adjusted life years (DALYs) is extremely high. The epidemiological analysis of the global burden of AMR in 2019 estimates 5 million deaths associated with AMR infections [5], which represents a signal that an overlooked pandemic driven by bacteria is among us: AMR.

## Bacterial pandemics throughout history

Pandemics are generally considered to be caused only by viruses, but history has proven this is not true. Pandemics that have remarkedly shaped the course of civilization have been caused by bacteria, such as the case of *Yersinia pestis* and *Vibrio cholerae*.

*Y. pestis* causes plague, which is transmitted between animals via their fleas. As it is a zoonotic bacterium, it can also be transmitted from animals to humans by the bite of infected fleas, through direct contact with infected materials, or by inhalation. According to the route of infection, it can manifest in three different forms: bubonic, septicemic, and pneumonic, respectively [6].

Bubonic plague is the most common. Following a flea bite, *Y. pestis* travels to the lymph node to replicate, causing inflammation, and, in advanced infections, lymph nodes can turn into suppurating open sores. Septicemic plague occurs when *Y. pestis* reaches the bloodstream following flea bites or through direct contact with infected materials through the skin, causing systemic infections. Septicemic plague can also be achieved at advanced stages of bubonic plague. Pneumonic plague is the least common but most lethal form of plague, usually caused by the spread of *Y. pestis* to the lungs at advanced stages of bubonic plague or from aerosolized infective droplets from infected patients. If left untreated, plague could have a case-fatality ratio of 30–100%, particularly in its septicemic and pneumonic forms [6].

Plague has been responsible for widespread pandemics throughout history. Between 541 and 543, it killed about 100 million people in the Roman empire, particularly in Constantinople. It was facilitated by the highly developed structure of trade and military routes and may have well led to the weakening and fall of the Byzantine empire. Following the initial pandemic, intermittent outbreaks occurred every 8 to 12 years for two centuries and then disappeared for unknown reasons [7]. A second pandemic, called the Black Death, was brought to Europe through the land and sea trade routes of the Silk Road and wiped out an estimated 15 to 23.5 million Europeans, representing about one-fourth to one-third of the population between 1347 and 1351. In cities like Genoa, in Italy, about half of the population died [7, 8]. The third (and current) pandemic probably originated in the Chinese province of Yunnan around 1855 and spread to the southern coast of China by heavy troop traffic. By reaching Hong Kong and Canton in 1894 and causing great epidemics, the disease was quickly disseminated all over the world. In India alone, a total of 12.5 million Indians are estimated to have been killed by plague between 1898 and 1918 [8].

Cholera is an acute diarrheal infection caused by the water-borne bacterium *V. cholerae* through the ingestion of contaminated food or water [9]. Following ingestion, *V. cholerae* produces the cholera toxin, which is responsible for a rapid and massive loss of body fluids leading to dehydration, hypovolemic shock, and death [7]. During the nineteenth century, cholera spread across the world from its original reservoir in the Ganges delta in India [9]. Six subsequent pandemics killed millions of people across all continents. The seventh (current) cholera pandemic is the most extensive in terms of geographic spread and duration [10].

## Near-miss pandemics

The wider spread of several infectious diseases to many geographic areas as a result of the acquisition of drug resistance, poor sanitation, climate changes, and increased human mobility and travels suggests that AMR can be considered a silent pandemic [7]. It is commonly not considered as such, since it has a less immediate impact on daily life compared to classical pandemics (e.g., COVID-19), but it can have potentially much higher negative impacts in the long term.

The line that separates an outbreak from a pandemic is very thin. Enterohaemorrhagic *Escherichia coli* (EHEC) represents an example of how quickly bacteria may evolve and how fast we should be to detect, identify, and characterize the pathogen to avoid spread and propose a remedy. The most severe complications of EHEC infections are the hemorrhagic colitis (bloody diarrhea) and the hemolytic uremic syndrome (HUS) which is a life-threatening disease caused by kidney damage. EHEC are Shiga toxin producers and causes outbreaks every year in many places in the world with a high incidence of HUS and even deaths. In 1996, the biggest recorded outbreak was in Japan and included over 8000 reported cases. In 2011 in Germany, the EHEC serotype O104:H4 caused the largest outbreaks by a food-borne pathogen with an incidence never seen before for an outbreak in Germany or worldwide, with 3000 cases of acute gastroenteritis, 855 of which developed HUS. It was characterized by high case fatality, of 1.1 cases per 100,000 inhabitants in 2011 versus an average of 0.1 cases per 100,000 inhabitants in 2001–2010, with 55 people who died from the infection. It also affected visitors from 15 other countries and was linked to a smaller subsequent outbreak in France [11]. It was later confirmed that EHEC acquired a strong adhesion capacity from another *E. coli* pathotype, called Enteroaggregative *E. coli* (EAEC), and increased antibiotic resistance, leading to a lineage with a strong ability to attach to host epithelial cells, infect, and resist first-line antibiotic treatments, including third-generation cephalosporins, although treatment with antibiotics was not recommended considering they may release the Shiga toxin and increase disease severeness [12].

The outbreak lasted for 2 months and was a major challenge for hospitals, public health, and food safety agencies. The Robert Koch Institute conducted a total of 13 epidemiological field investigations, using different study designs. Initially, lettuce, raw tomatoes, and cucumbers imported from Spain were identified as potential sources of the infection, and only later, the fenugreek sprouts, produced in Germany from seeds imported from Egypt, were identified as the vehicle causing the outbreak. Interestingly, it was not possible to detect the pathogen in sprouts consumed by the patients, highlighting the uniqueness of this outbreak [13]. It is still unclear how frequently sprouts are contaminated by EHEC, and this is of concern considering that sprouts are particularly vulnerable to bacterial contamination and are often consumed raw.

In summary, in about 2 months, the outbreak of pathogenic *E. coli* infection caused 3000 cases and 55 deaths. This very rapid increase in the number of cases in Germany, the serious illness, and the number of deaths shows how quickly an infectious bacterium can escalate to a major health threat. Although the resistance was only related to the first line of antibiotics, the increase in incidence and the challenges to stop infection demonstrate how dramatic an outbreak caused by an AMR pathogen might be. This outbreak is particularly indicative at reminding the importance of efficient surveillance and notification reporting systems, diagnostic tools, and the need for a close collaboration between the different players, doctors, scientists, health authorities, and food safety authorities.

## Silent pathogens of global AMR spread potential

Certain pathogens like Group A Streptococcus remain sensitive to penicillin despite the extensive and indiscriminate use 80 years after introduction. The reasons for that are still unknown, but it is believed that the circumstances favorable for the development of resistance have just not yet occurred [14].

The most plausible explanation is that β-lactamase, once produced, could be toxic to the microorganism and render it non-viable. Group A Streptococcus tolerant to penicillin have been isolated in the laboratory, but they have been associated with severe phenotypical defects (i.e., poor growth rates, morphological abnormalities, decreased M protein production) that could severely compromise bacterial fitness and survival in the human host, unless simultaneous acquisition of additional genetic elements could compensate for this defective phenotype [14, 15].

The fact that a resistance has not emerged so far does not mean it will not emerge in the future. Group B Streptococcus, similar to Group A Streptococcus, with increased resistance to penicillin, have been isolated in Japan [16, 17] and North America [18] due to amino acid substitutions next to conserved active-site motifs of penicillin-binding PBP2X [19–21]. Indeed, there have been several reports of the emergence of reduced susceptibility of Group A Streptococcus to penicillin in India [22] and Japan [23] and several reports of Group A Streptococcus isolates that are not sensitive or even resistant to β-lactam antibiotics in China [24]. In India, 8% of Group A Streptococcus clinical isolates were not sensitive to penicillin. It was reported an overall increase in penicillin minimum inhibitory concentration (MIC) ranging from 0.12 to 8 μg/ml. Moreover, the prevalence of resistance to cefotaxime, erythromycin, tetracycline, lev ofloxacin, clindamycin, and ceftriaxone was 4.2%, 83%, 51%, 8.9%, 40%, and 5.3%, respectively [25].

In the USA, two clinical Group A Streptococcus isolates showed eightfold higher MIC for ampicillin and amoxicillin (0.25 μg/ mL), and the MIC for cefotaxime (0.06 μg/mL) was threefold higher than for near-isogenic control isolates [26]. Both isolates had a PBP2x missense mutation (T553K) and a single-point mutation substitution within the topoisomerase subunit ParC (S79F). These results are at the susceptibility breakpoint for ampicillin resistance consistent with a first step in developing β-lactamase resistance [26]. Strikingly, there were no differences in growth rates between the two PBP2x T553K substitution mutants and the three closely related control strains [26]. Authors suggested that considering the close relatedness between the two T553K substitution mutants, the PBP2x mutation may not have arisen independently and that single nucleotide polymorphism differences within the emm cluster may indicate that the PBP2x mutation has emerged within 1–2 years prior to isolation.

An analysis of over 9667 Group A Streptococcus isolates to identify the relative frequency of PBP sequence variation showed that mutations on Group A Streptococcus PBPs (PBP2x, PBP1a, PBP1b, and PBP2a) were infrequently within strains included in the database [27]. In a similar study, 137 strains were identified with a mutation in PBP2x that caused a decreased susceptibility to some β-lactam antibiotics, including the commonly used penicillin G. Interestingly, these polymorphisms arose independently as a consequence of convergent evolution, presumably due to selection following exposure to a β-lactam antibiotic [28].

Considering the high incidence of Group A Streptococcus infections every year, accounting for approximately 800 million cases of sore throats and skin infections every year [29], increasing antibiotic resistance among Group A Streptococcus isolates is very concerning. In India, erythromycin resistance was found in 53% of isolates with inducible macrolide, 33% of isolates resistant to clindamycin, and 58% of isolates resistant to tetracycline. Erythromycin and tetracycline co‑resistance was found in 39% of tested Group A Streptococcus isolates, which has been attributed to the over‑prescription and use of these antibiotics [30]. In China, fluoroquinolone resistance is emerging, accounting for 1.3% of clinical isolates. Among them, 80% are also resistant to tetracycline and erythromycin. In China, fluoroquinolones are the third most commonly prescribed antibiotic and are a therapeutic alternative for multidrug-resistant Group A Streptococcus [31].

Although Group A Streptococcus is believed to be a strict human pathogen, the potential zoonotic potential of this pathogen has been broadly dismissed. Group A Streptococcus has been reported as the causative agent of a broad spectrum of diseases in animals. Most recently, among 115 pets presenting respiratory illnesses, Group A Streptococcus was isolated from 11 pets. Among them, 8 isolates were resistant to penicillin, macrolide, lincosamid, and tetracycline [32].

The capacity of hypervirulent Group A Streptococcus to acquire new tools, disseminate, and persist worldwide has already been demonstrated [33, 34] and recently culminated in the increase of scarlet fever in children and invasive Group A Streptococcal disease in all ages in the UK [35]. A scenario where Group A Streptococcus acquires resistance to penicillin would be catastrophic.

## AMR burden today

Epidemiological data allow us to deeply understand the impact of AMR on mankind and show the dramatic consequences it can have on human life. The best comprehensive study providing an overall precise picture of the AMR worldwide in 2019 is the Global Burden of Disease (GBD) study [5]. Thus, we refer to this study to understand the AMR phenomenon as a whole.

The GBD study estimated that, in 2019, 4.95 million (3.62–6.57, 95% UI) deaths were associated with bacterial AMR globally (on the basis of an alternative scenario of no infections). The 1.27 million deaths in 2019 directly attributable to AMR are the same as the combination of HIV (680,000) and malaria (627,000) deaths worldwide, and behind only COVID-19 and tuberculosis in terms of global deaths from an infection. At the same time, also the morbidity associated with AMR was shown particularly high, with 2.29 million (1.52–3.45, 95% UI) years lived with disability (YLD). The morbidity is a relevant parameter to be taken into account, as it has a relevant socio-economic impact and drains resources from countries to take care of the sequelae caused by diseases. This aspect is particularly critical for low and middle-income countries (LMICs) and can become a hurdle for their social and economic development. The GBD (considering both mortality and morbidity) associated with AMR was evaluated in 192 million (146–248, 95% UI) DALYs.

Looking at the rate of AMR-associated deaths per 100,000 all-cause deaths, the high-income countries (HICs) showed 55.7 (40.1–76.0, 95% UI) cases and Central Europe-Eastern Europe-Central Asia 67.7 cases (45.4–96.6, 95% UI), whereas Sub-Saharan Africa and South Asia 98.9 (78.6–124.2, 95% UI) and 76.8 (57.2–101.2, 95% UI) cases, respectively. Consistently, the rate of AMR-associated DALYs per 100,000 all-cause DALYs was 946.7 (649.8–1327.2, 95% UI) for HICs and 1826.9 (1274.5–2545.4, 95% UI) for Central Europe-Eastern Europe-Central Asia, whereas it was 6143.9 (4802.8–7792.2, 95% UI) in Sub-Saharan Africa and 3318.1 (2532.9–4291.7, 95% UI) in South Asia. There is a substantial higher burden of AMR-associated diseases in LMICs, which is obviously the consequence of the socio-economic disparity from several perspectives (hygiene, diagnostics, treatments, nutrition, healthy lifestyle), but it is also a sign of a reduced access to antibiotics.

The three infection syndromes accounting for the majority of the AMR-associated deaths and DALYs globally were lower respiratory tract, bloodstream, and intra-abdominal infections. The top 10 pathogens as etiological agents of AMR deaths at the global level were (by order of death numbers) as follows: *Escherichia coli, Staphylococcus aureus*, *Klebsiella pneumoniae*, *Streptococcus pneumoniae*, *Acinetobacter baumannii*, *Pseudomonas aeruginosa*, *Mycobacterium tuberculosis*, *Enterococcus faecium*, *Enterobacter* spp., and Group B Streptococcus. The 10 top etiological agents for AMR-associated DALYs were (by order of DALY counts) as follows: *S. pneumoniae*, *E. coli*, *K. pneumoniae*, *S. aureus*, *P. aeruginosa*, *A. baumannii*, *Salmonella* Typhi, Group B Streptococcus, *M. tuberculosis*, *Enterobacter* spp. Among all these pathogens, vaccines are available only for *S. pneumoniae*, but, despite this, pneumococcal infections represent the fourth cause of AMR-associated deaths in the world.

The three pathogens representing, in the HICs, the major fractions of etiological agents of AMR-associated deaths were (by order of prevalence) *S. aureus*, *E. coli*, and *S. pneumoniae*, whereas those three for Sub-Saharan Africa were (by order of prevalence) *S. pneumoniae*, *K. pneumoniae*, and *E. coli*. For AMR-associated DALYs in HICs, the three most prevalent pathogens (by order of prevalence) were *S. aureus*, *E. coli*, and *K. pneumoniae* whereas in sub-Saharan Africa were still *S. pneumoniae*, *K. pneumoniae*, and *E. coli*. The fact that these pathogens can cause infections of airway tissues or sepsis matches perfectly with the observation that lower respiratory tract and bloodstream infections generate a substantial proportion of the AMR-associated deaths and DALYs.

Although the WHO launched, in 2015, the Global Action Plan to tackle AMR and the Global Antimicrobial Resistance and Use Surveillance System (GLASS) project to improve the reporting of AMR cases and their etiological agents, an important gap in data on AMR still exists for LMICs [36, 37]. However, according to the GLASS project, the case-reporting capacity of countries involved in the GLASS project increased substantially from 2016 to 2020 [38].

The annual epidemiological report for 2021 of the European Antimicrobial Resistance Surveillance Network [39] shows that, between 2019 and 2021, the number of reported AMR isolates increased substantially for most of the pathogens (despite the reporting of cases of AMR pathogens was affected by the COVID-19 pandemic). For example, in 2021, the number of isolates increased by 7.2% compared to 2020, for all the bacteria under surveillance. However, comparing the number of AMR isolates in 2021 with the average of those between 2018 and 2019, the largest increases were detected for *Acinetobacter* spp. (+ 73.9%), *E. faecium* (+ 32.5%), and *E. faecalis* (+ 11.7%). There was almost no change in *K. pneumoniae* (+ 0.03%) and *P. aeruginosa* (− 0.9%), and a decrease for *S. aureus* (− 5.5%), *E. coli* (− 11.8%), and particularly *S. pneumoniae* (− 45.6%). Looking at the data with more granularity for specific antibiotics, important differences can be detected among the diverse types of drugs. Nevertheless, a significant increase in the number of reported isolates resistant to several antibiotics between 2017 and 2020 has been observed for most of the critical pathogens.

In 2021, the most commonly reported bacterial species in AMR diseases within European countries were *E. coli* (39.4% of all reported cases), *S. aureus* (22.1%), and *K. pneumoniae* (11.9%) [39].

According to the US Centers for Disease Control and Prevention (CDC) special report on AMR during the COVID-19 pandemic [40], the AMR cases and deaths caused by pathogens identified as urgent or serious threats are increasing for most of them, overall or as the hospital-onset. For example, the deaths caused by carbapenem-resistant *A. baumannii* displayed an overall increase of 35%, whereas the deaths caused by carbapenem-resistant Enterobacterales displayed a hospital-onset increase of 35%.

## Tools to combat and prevent AMR

**Fig. 1:**
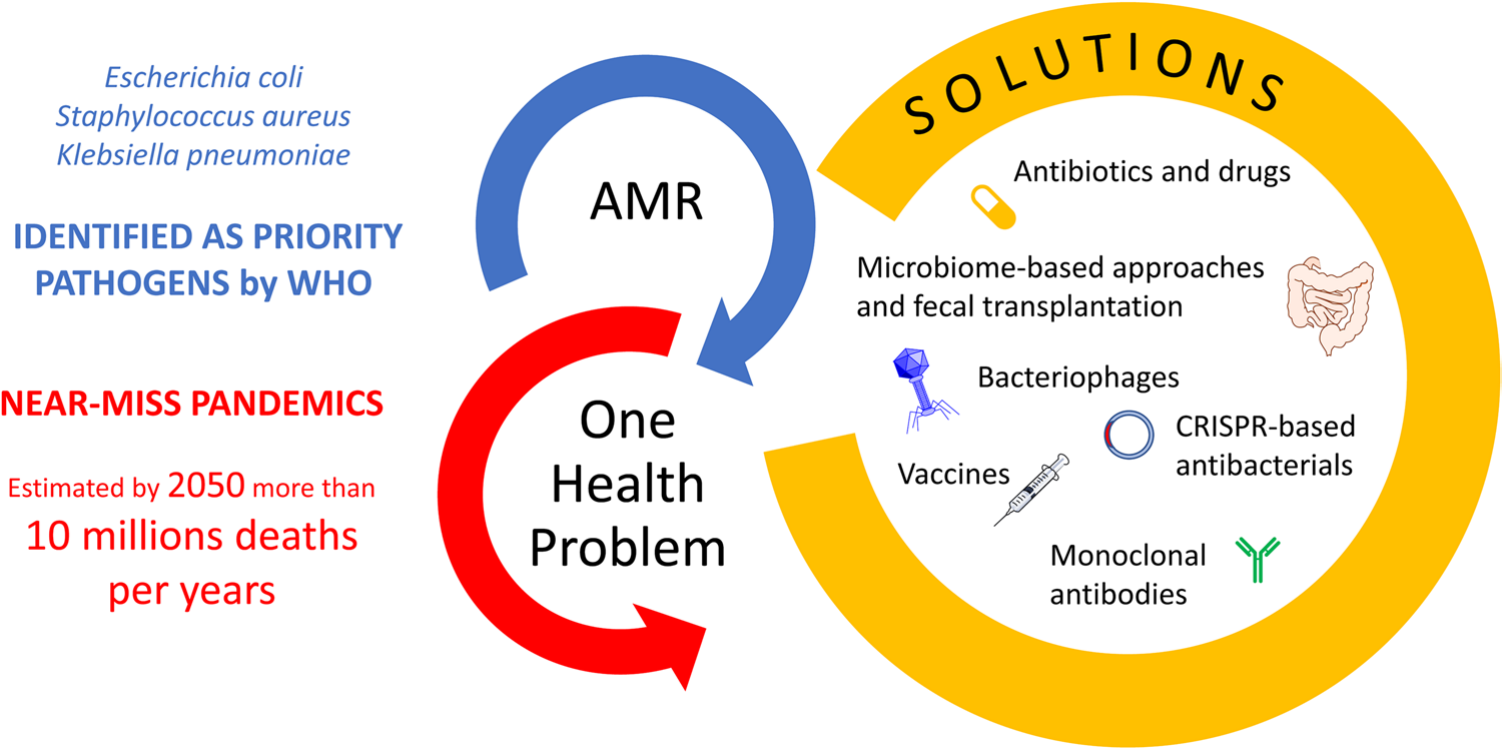
Tools to combat and prevent AMR

AMR is an unavoidable consequence of the use of antimicrobials. Resistance generally arises within 10 years from the clinical introduction. Therefore, there is the need to build an arsenal of antibacterial agents with different targeting capabilities able to slow the emergence of resistance and its spread (Fig. 1).

### Antibiotics and drugs

There is the need to develop new classes of antibiotics that work through novel mechanisms. Among the new targets being explored, there are efflux pumps and biofilm or combination therapies targeting both, essential bacterial functions and factors contributing to resistance, or peptides with potential antibiotic properties identified by artificial intelligence on the human proteome. An important line of research is also the identification of novel antibiotic compounds from nature, including soil, as in the case of malacidins [41], which are reported to kill many multidrug-resistant pathogens and to act on the bacterial cell wall in a unique way compared to other existing calcium-dependent antibiotics.

### Microbiome-based approaches and fecal transplantation

Non-traditional approaches to treating AMR infections include live microbiome-based therapeutic products. There is much evidence that “good” bacteria can help to eliminate “bad” bacteria. In a study in Thailand on 200 volunteers living in a rural area, volunteers containing *Bacillus subtilis* in fecal samples were not colonized by *S. aureus* [42], providing evidence of the key role that microbiome may play in protecting from colonization. Studies are ongoing to evaluate whether a probiotic product that contains only *B. subtilis* can eliminate *S. aureus* in humans and can be used to reduce MRSA infection rates in hospitals [43].

The rationale of fecal microbiota transplantation (FMT) is that the normal microbiota in the gut protects the gastrointestinal mucosa against microbial pathogens providing “colonization resistance,” and stopping the overgrowth by invading microorganisms. Although the use of FMT dates back to fourth-century China, only in 2000, after a *Clostridium difficile* epidemic caused by hypervirulent strains, BI/NAP1/027, associated with higher rates of infection, increased mortality, resistance to fluoroquinolones, and increased toxin production, it became a popular treatment. The fecal transplantation is based on putting stool that has been pre-screened for infectious agents and antibiotic-resistant organisms from a healthy donor in the colon of an infected patient in order to restore a healthy and diverse gut microbiome. This therapy, also called bacteriotherapy, was immediately shown to be highly effective and is used today as a routine therapy to treat recurrent *C. difficile* infections [44].

### Bacteriophages

Bacteriophages are viruses that parasitize, infect, and replicate within bacteria. They are specific to the bacteria they infect and can kill them without harming other cells. Following binding, they enter the bacterial cell, replicate, and cause bacterial lysis. The phage therapy is considered a very promising alternative for bacterial infections. However, due to the lack of data from randomized trial, as well as several regulatory issues, phages are only used in complex cases for patients in therapeutic failure and are always combined with antibiotic treatment, although they may be an effective alternative to antibiotics. They may potentially control infection by resistant pathogens, and limiting the use of antibiotics may limit the emergence of new resistant strains. The potential applications may be not only in human health but also in animal health and in the environment [45, 46]. Further studies will be needed to evaluate efficacy, safety, and ability to induce bacterial resistance.

### CRISPR-based antibacterials

Another innovative approach for new antimicrobials is based on CRISPR-Cas, a novel and adaptable method capable of targeting any pathogenic bacteria. CRISPR-Cas plays a key role in the defense mechanism of bacteria and archaea by the insertion of foreign DNA into the chromosome, such as mobile genetic elements or bacteriophages. The system consists of an endonuclease, Cas9, which is guided to the specific genomic loci by a single-guide RNA containing complementary base pairs where it induces a double-stranded DNA (dsDNA) break. CRISPR-Cas has been extensively explored to selectively insert, delete, or mutate genes in any species. One of the key features of CRISPR-Cas is the high sequence specificity which allows to discriminate between pathogenic or commensal bacterial species. Therefore, the CRISPR-Cas machinery may be used to attack rather than defend bacteria, and CRISPR-guided RNAs can be designed to target either virulence or essential chromosomal genes specific to pathogens. Induction of a double-stranded DNA break can result in bacterial death if induced at the chromosomal level, therefore CRISPR-Cas9 system may be a potential antibacterial gene-editing agent [47]. One effective way to deliver the CRISPR-Cas system is via engineered phage-based vectors, which can also be considered as a phage-derived antimicrobial therapy [48]. The use of phage-based vectors to deliver the CRISPR-Cas system into target bacteria has still several limitations related to a narrow host range, bacterial resistance, safety issues, and phage clearance.

### Vaccines

The main challenge of most of the resistant bacterial pathogens is their high antigenic variability. Therefore, vaccines able to prevent AMR infections are expected to be multicomponent. Conjugation of polysaccharides to carrier protein generates potent vaccine antigens able to induce a strong T-cell dependent response, high-avidity antibodies, and immune memory [49]. Vaccines against pneumococcus are very effective in preventing the disease; however, high multivalency is needed to cover the majority of pneumococcal serotypes. Increasing the uptake of existing pneumococcal vaccines globally would reduce infections caused by drug-resistant strains.

The repeating unit of the polysaccharide can be also chemically synthetized, as in the case of a *Shigella* vaccine which is now in clinical trials [50]. *E. coli* strains are engineered to produce bioconjugated polysaccharide vaccine antigens, in which both the polysaccharide and the carrier protein are synthetized in *E. coli* and conjugated by the PglB oligosaccharyltransferase enzyme [51, 52]. Bioconjugates against AMR pathogens such as *Shigella*, extraintestinal pathogenic *E. coli*, and *K. pneumoniae* are in development [51, 53, 54]. A novel approach is based on the MAPS (multiple antigen-presenting system) that can potentially include multiple polysaccharides and proteins in the same complex.

Genetic engineering is also used to generate rationally designed attenuated bacterial strains. This approach involves the identification of bacterial functions, which do not impact the ability of the strain to colonize and replicate in the host to induce an effective immune response while impacting the strain’s ability to cause disease. This approach has been extensively applied to enterotoxigenic pathogens such as *Salmonella*, *Vibrio cholera*, and *Shigella* strains [55]. Although very promising, no vaccines based on this technology have been licensed so far. Gram-negative bacterial strains can also be engineered to produce high yields of outer membrane vesicles (OMVs). By chromosomal mutations, strains with an “overblebbing” phenotype and expressing a less reactogenic lipopolysaccharide (LPS) can be generated. OMVs derived from these recombinant strains, also named GMMA (generalized module membrane antigens), can be the ideal delivery system for protective antigens such as the O-antigen of *Shigella* and *Salmonella* and can further engineered to over-express heterologous antigens [56]. *Shigella* and *Salmonella* GMMA–based vaccines are currently in clinical development.

The genomic era has changed the vaccine landscape even more. Since the first genome sequence of a bacterial pathogen, *Haemophilus influenzae* type B, the number of sequenced genomes available in public databases is of the order of a hundred thousand. The genome represents a catalogue of all antigens that are potentially expressed at all stages of the bacterial virulence cycle. This has allowed, through “in silico” analysis, the identification of antigens and the discovery of a breakthrough approach referred to as “reverse vaccinology.” The first success of this approach has been the development of the multivalent meningococcal B vaccine, named 4CMenB, which is now registered in more than 45 countries worldwide [57]. Interestingly, retrospective analysis has shown the ability of the 4CMenB vaccination to reduce gonococcal infections [58]. This evidence is of particular importance considering the high rate of antimicrobial resistance in gonococcal strains.

With the access to genomic information of any pathogen, we have entered the era of the “digital vaccine” in which in every laboratory, even before accessing the pathogen, the gene encoding for antigens of interest can be identified, synthetized and delivered as DNA or mRNA [59], or cloned in viral vectors like poxvirus, alphavirus, adenovirus, vesicular stomatitis, and measles virus [60]. mRNA and adenovirus-based vaccines have been recently used in large immunization campaigns and proven to be protective against COVID-19. These technologies have been applied to a variety of viral and bacterial pathogens, and many preclinical and clinical studies have been completed or are ongoing, such as an adenoviral-vectored vaccine against group B meningococcal disease [61], a single-dose mRNA-LNP vaccine against lethal plague [62] and mRNA delivery of dimeric human IgA to reduce *Salmonella* enterica serovar Typhimurium invasion in the intestine and *P. aeruginosa* colonization in the lung [63]. It will be important to know how much these new technologies will apply to vaccines against AMR pathogens, known to be much more complex than SARS-Cov2.

As known, for SARS-Cov2, one of the main challenges is the sequence variation in the S antigen and the fast evolution in generating variants. This aspect will represent a challenge in the development of vaccines against AMR, for the majority of which the known and most abundant surface antigens are known to be also the most variable. Nevertheless, the access to multiple genome sequences providing data on strain-to-strain sequence variations of specific antigens, the knowledge and/or prediction of the three-dimensional structure, and the identification of the main protective epitopes may allow the design of recombinant antigens more immunogenic and more cross-protective. This multifactorial approach, named “structural vaccinology” or “reverse vaccinology 2.0” [64], has proven to be successful with the RSV (respiratory syncytial virus) vaccine, based on a stable and strongly immunogenic “pre-F” antigen [65], which is the basis of the recently licensed RSV vaccine by GSK [66]. Isolation of human monoclonal antibodies from sera of convalescent subjects can allow the in-depth characterization of antibody response following infection, the identification of the most immunogenic and protective epitopes, and even the identification of new protective antigens.

Antigens can be also expressed on nanoparticles to increase immunogenicity and stability. The presentation of multiple copies of the antigen on nanoparticles may enhance the B cell response, whereas the nanoparticle structure may facilitate the uptake by the antigen-presenting cells, leading to a more efficient antigen presentation to T cells. Examples of self- assembling nanoparticle vaccines are the virus-like particles, as in the case of the Hepatitis B and Papilloma virus vaccines. More recently, computationally designed nanoparticles have been proposed as a robust and versatile platform for antigen presentation [67].

Finally, the discovery and licensure of new adjuvants have impacted enormously the vaccine field. Highly purified antigenic determinants of a vaccine are often poorly immunogenic at the same time. Adjuvants enhance the antigen immunogenicity by inducing more potent humoral and/or cell-mediated immunity. Adjuvants can also promote a better quality of the immune response, for example by improving the functionality of the humoral immune response or amplifying the memory compartment that can be translated into a more efficacious immunization. In addition, adjuvants may shorten the time that the body is able to mount the immune response, increase the breadth of the immunity against different strains of a pathogen, promote the dose-sparing of the vaccine, and reduce the number of vaccine doses to be administered to achieve protection from the pathogen [68–70]. Adjuvants work mainly by stimulating cells of the innate immune system or at the interface between innate and adaptive immune systems, but they can also stimulate directly lymphocytes [68, 69, 71, 72]. Research and development on novel adjuvants are continuously growing because more potent adjuvants can really be transformative in the struggle against infectious disease, by leading to the production of new and effective vaccines. One clear example is Adjuvant System 01 (AS01), which is a liposome-based adjuvant containing the saponin QS-21 and the MPL (a TLR4 agonist), able to strongly stimulate innate immunity and induce both potent antigen-specific antibody response and IFN-g CD4 T cell response [73]. AS01 made possible a novel Herpes zoster vaccine with 95% efficacy [74], the first malaria vaccine [75], and the first RSV vaccine for older adult with an efficacy of about 95% [66].

### Monoclonal antibodies

Monoclonal antibodies (mAbs) are an important therapeutic tool to fight AMR [76]. Although antibodies are successfully used to treat autoimmune disease and cancer, their application in infectious disease is still very limited. One of the limitations is the complexity of bacterial pathogenesis and bacterial evolution resulting in the need to target multiple antigens to impact multiple virulence steps. mAbs targeting different epitopes can be combined and act synergistically or can be used in combination with antibiotics or alternative therapies. The majority of human mAbs in development target mainly nosocomial bacterial pathogens such as *S. aureus*, *P. aeruginosa*, *C. difficile*, and *A. baumannii*. Many other human mAbs against additional AMR pathogens are being investigated at the preclinical level. All these monoclonals target individual pathogens, with the exception of a mAb targeting a DNA binding protein (DNABII), a protein playing a key role in biofilm formation and conserved among multiple bacteria. If successful, it will be the first monoclonal antibody with pan-activity [77]. One drawback of human mAbs is the high cost. However, the new technologies are allowing the isolation of antibodies with much higher affinity which can be used at lower dose and with a lower production cost, rendering the use of human mAbs for infectious disease more affordable.

## Conclusions

It has been estimated that by 2050, AMR could cause more than 10 million deaths per year [78]. If this trend will not be reversed, according to this prediction, the AMR will become the first cause of death worldwide [79]. Because of the AMR impacts on the health of animals and crops, it has been estimated that up to 24 million people could undergo extreme poverty by 2030 [80]. A significant portion of antimicrobials globally is also used in food-producing animals as prophylaxis or to promote faster growth [81], especially in LMICs [82, 83]. The antimicrobials used in agriculture, aquaculture, veterinary, and human healthcare are often the same [84, 85]. Therefore, the overuse and misuse of antimicrobials in human medicine, livestock, fisheries, and crop production have caused high AMR emergence and contributed to the development of resistance in humans. Therefore, it is of fundamental importance that the use of antimicrobials will become appropriate across the “One Health” universe including humans, animals, and environment [86].

The AMR issue has been typically addressed by searching and developing new antibiotics. Although many investments have been made in the research and development space, the development of new antibiotics is scientifically challenging and very expensive. Considering the rate of onset of resistance and the time needed for a new antibiotic to become obsolete, incentives will be needed for R&D to promote science independently by the investment returns from sale volumes. In this perspective, vaccines represent a very valid solution to prevent the spread of resistant pathogens and to reduce the incidence of disease and the need for antibiotic treatments. A recent review summarizes all vaccines which are already available and those that are in clinical or preclinical phases that can counteract the rise in antimicrobial resistance [87]. Interestingly, 61 new vaccines are in the development phase and 94 in the preclinical phase. This very high number of existing and potentially new vaccines shows how the advent of new technologies in the vaccine field is opening the way to the prevention of many infectious diseases and potentially to the reduction of AMR pathogens.

The implementation of global surveillance programs for the rapid detection of resistance and dissemination of antibiotic resistance will be critical to controlling the ongoing antibiotic resistance pandemic. An efficient surveillance health-system approach to monitor antibiotic drug use and antimicrobial resistance is urgently needed to monitor the resistance particularly in developing countries. Indeed, in 2015, WHO launched the Global Antimicrobial Resistance and Use Surveillance System (GLASS), the first global collaborative effort to standardize the AMR surveillance via the collection, analysis, and interpretation of data from countries [38]. GLASS is based on the strong commitment of participating countries and close collaboration with AMR regional networks. Cutting-edge technologies such as digital surveillance and next-generation sequences introduced for the COVID-19 experience could open the way to control AMR spreading. This is important considering that AMR microorganisms do not recognize “country borders,” and this is of particular concern in an era of globalization. Following travel to countries with high rates of AMR, travelers can become colonized by strains with new AMR genes. Travel to China, India, or Northern Africa increased the level of colonization of Swedish travelers with extended-spectrum β-lactamase producing Enterobacteriaceae from 2.4 to 68%, and then up to 1 year was needed to return to the levels before traveling [88]. One of the key genes for carbapenem resistance (NDM-1) was first isolated in India and then detected worldwide [89, 90]. The mcr1 gene that confers resistance to colistin was also first detected in China [91] but then has been found worldwide [92].

**Fig. 2:**
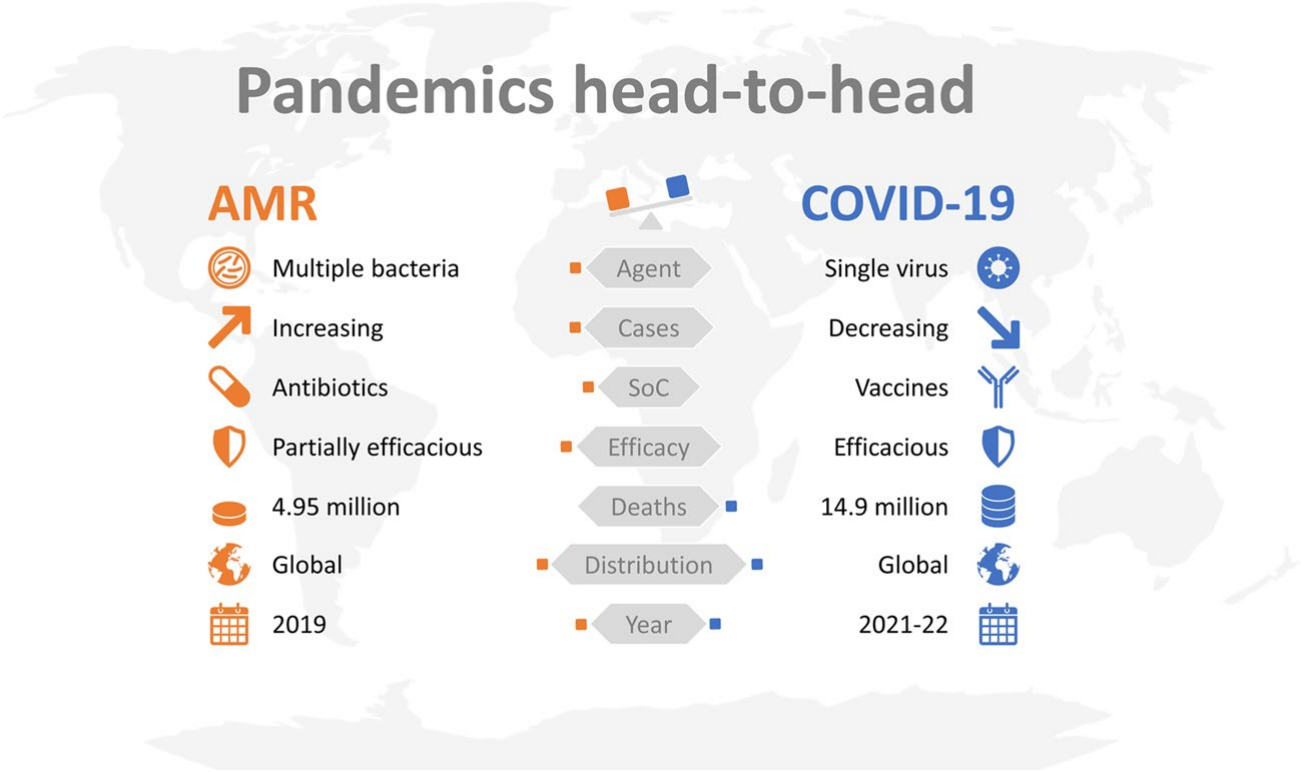
Head-to-head comparison between AMR and COVID-19

Despite the perception raised by COVID-19, which was estimated to cause 14.9 million (13.3–16.6) deaths between 2021 and 2022 [93], bacteria can also be responsible for pandemics with devastating outcomes. Plague and cholera pandemics disseminated along trade and military routes and the speed at which new pathogens and infectious diseases are disseminated around the world have only accelerated throughout history. In comparison to COVID-19, which was caused by a single microorganism and vaccines that are currently available, AMR is caused by a broad spectrum of very distinct pathogens, and the trend is on the rise due to the absence of broadly protective strategies (Fig. 2).

The estimate of 4.95 million (3.62–6.57) deaths associated with bacterial AMR globally in 2019 by the GDB study is already of serious concern, but the trend of AMR data indicates that these numbers can increase dramatically in the future. Unfortunately, public awareness and concern about AMR are disproportionately lower compared, for example, to the COVID-19 pandemic or to cardiovascular diseases or cancers, despite the evidence indicating that it is already a threat, with the potential to become in the near future the most important health problem worldwide.
